# D-Cycloserine in Neuropsychiatric Diseases: A Systematic Review

**DOI:** 10.1093/ijnp/pyv102

**Published:** 2015-09-12

**Authors:** Sebastian Schade, Walter Paulus

**Affiliations:** University Medical Center, Georg-August University, Department of Clinical Neurophysiology, Robert-Koch Straße 40, 37075 Göttingen, Germany.

## Abstract

D-Cycloserine, known from tuberculosis therapy, has been widely introduced to neuropsychiatric studies, since its central active mechanism as a partial NMDA-agonist has been found. In this review, we evaluate its therapeutic potential in neuropsychological disorders and discuss its pitfalls in terms of dosing and application frequency as well as its safety in low-dose therapy. Therefore, we identified 91 clinical trials by performing a Medline search. We demonstrate in part preliminary but increasing evidence that D-cycloserine may be effective in various psychiatric diseases, including schizophrenia, anxiety disorders, addiction, eating disorders, major depression, and autism as well as in neurological diseases, including dementia, Alzheimer’s disease, and spinocerebellar degeneration. D-Cycloserine in low-dose therapy is safe, but there is still a need for new drugs with higher specificity to the different N-methyl-D-aspartate-receptor subunits.

## Introduction

D-Cycloserine (DCS) has a unique potential to target the glycine-binding site of N-methyl-D-aspartate (NMDA) receptors in humans. Alternative drugs applicable in human as well as in animal experiments are summarized in Table 1. DCS is a natural product of *Streptomyces orchidaceus* and *Streptomyces garyphalus*, which has been known in tuberculosis therapy since the late 1950s (Offe, 1988). Years later, Thomas et al. (1988) found its central active mechanism to be a selective partial NMDA agonist acting at the glycine-binding site of the NMDA receptor. It was postulated, and later proven on slice preparations, that DCS influences long-term potentiation (LTP), a neuronal mechanism thought to be relevant for learning (Watanabe et al., 1992). Since then, neuropsychiatric studies have been carried out to evaluate the potential of DCS for neurological and psychiatric conditions such as Alzheimer’s disease, schizophrenia, depression, and anxiety disorders. Even though DCS has already been approved by the U.S. Food and Drug Administration for human use (in tuberculosis therapy and some urinary tract infections), most research data on neuroplasticity is still preliminary, and some results are even heterogeneous. Here we review the literature investigating the therapeutic potential of DCS, especially in terms of its scientific and therapeutic potential, as well as its safety issues.

**Table 1.** Agonists and Antagonists of the Glycine B Site of the NMDA-Receptor

**Agonists**		**Antagonists**
Full natural agonists	Glycine	Kynurenic acid and derivates (e.g. 5,7-diCl-KYN)
	(R)-alanine	2-Carboxyindoles (e.g. L-689,560)
	(R)-serine	2-Carboxytetrahydroquinolines
Partial agonists	ACPC (1-Aminocyclopropane carboxylic acid)	4-Hydroxy-2-quinolones
	ACBC (1-Aminocyclobutanecarboxylic acid)	Quinoxaline-2,3-diones
	Cycloleucin	3-Hydroxy-1H-1-benzazepine-2,5-diones
	D-Cycloserine	Tricyclic glycineB site antagonists
	HA-966 and derivates (e.g. L-687,414)	

For detailed information on different substances, see Danysz and Parsons (1998)

## Neurophysiological Aspects

**Figure 1.**
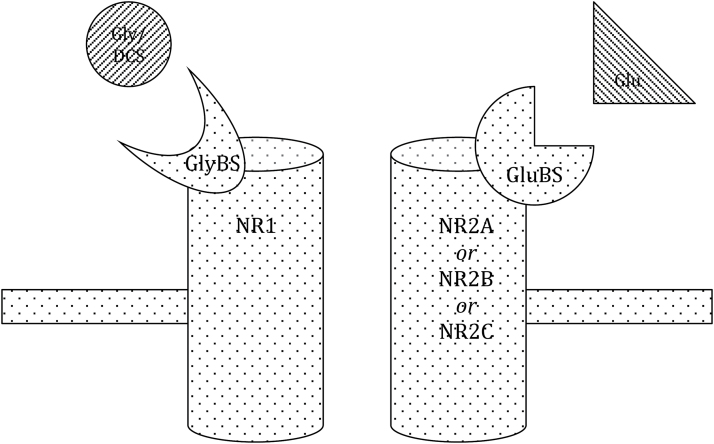
Schematic structure of the NMDA receptor. The NMDA receptor consists of 2 subunits (NR1 and NR2A, NR2B or NR2C). The NR2 subunits hold the glutamate binding site (GluBS), where the main agonist glutamate (Glu) binds. The NR1 subunit holds the glycine binding site (GlyBS), where the natural co-agonist glycine (Gly) or the partial agonist D-Cycloserine (DCS) bind. For further agonists and antagonists of the GlyBS see Table 1. For further details on the structure of the NMDA receptor see Dravid et al. (2010).

The NMDA receptor plays a crucial role in cortical neuroplasticity through its mechanism called LTP. This has been proven to be relevant for various learning processes (Watanabe et al., 1992) and thereby gives a strong rational for NMDA receptor influencing drugs (like DCS; see Table 1) to be used in illnesses that are based on deficits in neuroplasticity (eg, dementia) and/or therapies that rely on learning processes (eg, fear exposure therapy). The NMDA receptor consists of 2 subunits: NR1 and NR2 (Figure 1). DCS acts at the glycine-binding site of the NMDA receptor, which is located at its NR1 subunit. In contrast to the natural binding substance, glycine (as well as D-alanine and D-serine), DCS acts as a partial agonist (Watson et al., 1990), meaning that in vivo, it acts like an agonist at low doses but has antagonistic features with high doses. This seems to be due to its different receptor subtype selectivity and intrinsic action, which depends on various NR2 subunits (NR2A, NR2B, NR2C), the location of glutamate binding (Dravid et al., 2010). Presumably, the effects seen in vivo at low doses of DCS reflect its agonistic action at the NR1/NR2C receptors, for which it has a high affinity, while at high doses the effects might be due to antagonistic inhibition of NR1/NR2A and NR1/NR2B receptors, for which DCS has a lower affinity (Danysz and Parsons, 1998).

When targeting NMDA receptors that consist of NR2C subunits, DCS produces a 200% depolarization (compared with glycine) that is not pH-sensitive and seems not to depend on concentrations of glycine (Sheinin et al., 2001). NR2C units are mainly expressed in cerebellar structures, but are also found in the striatum, hippocampus, olfactory bulb, retrosplenial cortex, thalamus, pontine, and vestibular nuclei (Karavanova et al., 2007). NR2C knockout mice show deficits in tests of fear acquisition and working memory, implying that NMDA receptors consisting of NR2C subunits play a substantial role in fear learning processes (Hillman et al., 2011). In healthy animals, DCS leads to a better extinction of conditioned fears (Walker et al., 2002; Ledgerwood et al., 2003), enhances consolidation and retrieval of memories (Quartermain et al., 1994), and improves visual recognition memory (Matsuoka and Aigner, 1996). Interestingly, facilitation of the fear extinction process works only once, whereas the retrieval of a previous extinction memory (reextinction) seems not to be influenced by DCS (Langton and Richardson, 2008). With repeated applications, a reduction in efficacy has been shown in chronic administration of DCS in animals (Quartermain et al., 1994). A mechanism of endocytosis of the receptor has been hypothesized (Nong et al., 2003), according to which a single dose of DCS, but not repeated doses, may improve cognition in humans (Goff et al., 2008). Furthermore, a meta-analysis on exposure therapy showed that DCS is more efficacious when administered only a limited number of times (Norberg et al., 2008).

The mechanism of action of DCS might change or even reverse under conditions with great stress and might be due to different surrounding neurotransmitter concentrations (Hood et al., 1989; Watson et al., 1990; Sheinin et al., 2001; Davis et al., 2006). In line with this, DCS was shown to partially reverse the deficits of fear extinction learning due to sleep deprivation in (“stressed”) rats (Silvestri and Root, 2008). This, however, could not be reproduced in humans in a large multi-center study on the efficacy of cognitive behavioral therapy in correlation with sleep quality and DCS administration (Zalta et al., 2013). This study is probably not directly comparable, since subjects self-reporting a feeling of “not being rested” might not reflect sleep deprivation in rats in terms of stress. Nevertheless, the possible impact of stress on the efficacy of DCS should be taken into account when conducting trials with this substance in neuropsychiatric diseases, especially those that go along with sleep disturbances (eg, depression) or fear (eg, schizophrenia, anxiety disorders).

Altogether, DCS seems to have an impact on cognitive functions, mainly those associated with NMDA receptor-dependent mechanisms like LTP in learning processes. Part of this effect seems to be a stabilization of NMDA receptors, with a consequent facilitation of cortical neuroplasticity, as has been proven neurophysiologically via transcranial direct current stimulation (Nitsche et al., 2004; Kuo et al., 2008; Chaieb et al., 2012). Clinically, neurorehabilitation might benefit from DCS, since it improved functional recovery after cerebral damage in a mouse model (Adeleye et al., 2010).

## Safety Aspects

DCS has been used for over one-half a century in tuberculosis therapy, the doses used for this indication being clearly up to about 20 times higher than for modulation of neuroplasticity. The pharmacological properties and side effects are well known and have been described elsewhere (Goodman et al., 2001; Peloquin, 2008). To summarize, the maximum concentration (20–35 µg/mL) in blood is reached 2 hours after oral application of 250 to 500mg DCS. Half-life time varies from 8 to 12 hours depending on renal function. About 54% to 79% of oral intake reaches the cerebrospinal fluid. The typical application interval in antituberculosis therapy is 250 to 500mg twice daily (Peloquin, 2008). Side effects, mainly associated with high dosages, include hyperexcitability, dizziness, depression, anxiety, confusion, memory loss, and lethargy, as well as very rare seizures (especially with blood levels exceeding 35 µg/mL). Gastrointestinal trouble, rash, allergy, fever, and cardiovascular problems (including cardiac arrhythmia) are also described on rare occasions (Goodman et al., 2001; Peloquin, 2008).

The possibility of seizures has been investigated further. So far, animal studies report DCS to have more of an anticonvulsive effect than otherwise, especially in low-dose applications (Wlaz et al., 1994). As no changes were seen in the cerebral levels of neuronal amino acids, such changes are unlikely to be the cause of the rarely described convulsive side effects of DCS in humans (Baran et al., 1995).

We conducted a Medline search using the search item “D-cycloserine” with the filter of “Clinical trial” and “Human.” Studies up until December 2014 were included. The reference lists of the identified studies were examined for further studies. All studies were examined by the 2 authors of this review for applicability. In vitro trials, trials in tuberculosis therapy, as well as recalculations of former data were excluded by definition. Results can be found in supplementary Material. In 91 studies, more than 2100 patients were treated with DCS (mainly single doses or once weekly with a maximum dosage of 500mg/d) (for details on dosing, see supplementary Material). Frequent complaints of the DCS patients included psychopathological stimulation (eg, anxiety, euphoria, agitation, feeling “stimulated”), dizziness/drowsiness, fatigue, headache, and gastro-intestinal disturbance, similar to placebo controls (n>1500). Drop-out rates were also similar between verum and placebo. The highest drop-out rate was reported by Goff et al. (2005) while conducting a 6-month trial in schizophrenia patients with 50mg/d DCS coadministered with conventional antipsychotics. More detailed analysis of the subjects in this study did not show a difference between placebo and verum, reinforcing the theory that the high drop-out rate is not drug related but illness-associated, since long-term studies with schizophrenic patients often have high drop-out rates (Thompson et al., 2011).

When administering DCS in studies with patients, interactions with other drugs should be taken into account. For instance, antidepressants such as imipramine or citalopram can offset the facilitating effect on extinction of DCS in animals (Popik et al., 2000; Werner-Seidler and Richardson, 2007). Neuroleptics such as olanzapine and clozapine seem to impair the effects of DCS as well, especially in schizophrenic patients (Goff et al., 1996; Goff et al., 1999a; van Berckel et al., 1999).

## Therapeutic Implications

DCS is a typical example of translational research from neurobiochemical considerations to animal experiments and application in various neuropsychiatric diseases. First, the central acting mechanism of DCS was found (Thomas et al., 1988). DCS acts at the glycine-binding side of the NMDA receptor and thereby modulates its activity. It has been proven that NMDA receptors play a crucial role in neuroplasticity of the human brain through a mechanism called LTP (Watanabe et al., 1992). This led to the rationale that DCS might modulate neuroplasticity, which has been proven in humans by neurophysiological studies with transcranial direct current stimulation (Nitsche et al., 2004; Kuo et al., 2008; Chaieb et al., 2012). Neuroplasticity itself plays an important role in higher cognitive functions like learning and memory. Therefore, DCS might modulate these processes, which gave the rational of studying the effects of DCS on diseases associated with memory/learning deficits (eg, dementia, autism) on therapies that rely on learning processes (eg, exposure therapy in anxiety disorders or cue exposure therapy in addiction). In the following, various clinical applications of DCS in neuropsychiatric diseases will be outlined.

### Psychiatric Diseases

The rationale of DCS in psychiatric diseases like schizophrenia and depression is based on the glutamate hypothesis, although the exact mechanisms of the pathology are unknown. In schizophrenia, the fact that glutaminergic antagonists model symptoms of the disease and that genes of the glutaminergic system are associated with a higher risk of schizophrenia strengthens this hypothesis (Hashimoto et al., 2013). Since the NMDA receptor plays an important role in the glutaminergic system, its partial agonist DCS was speculated to influence symptoms of the disease. A pioneering group with Donald C. Goff in Harvard first investigated the therapeutic value of DCS in schizophrenia. Early studies showed a dose-dependent improvement in negative symptoms (affective flattening, alogia, avolition, anhedonia and attentional impairment) (Andreasen, 1982) in schizophrenic patients with best results at a dose of 50mg/d (Goff et al., 1995, 1999b; Heresco-Levy et al., 1998; Heresco-Levy et al., 2002). However, DCS in combination with conventional neuroleptics (clozapine, olanzapine) caused negative symptoms to worsen (Goff et al., 1996, 1999a; van Berckel et al., 1999), though further studies later showed no or only marginal effects of DCS on negative symptoms (Duncan et al., 2004; Goff et al., 2005; Buchanan et al., 2007). There might be different reasons for these seemingly contradictory results. Some could be due to the above-described, only partially agonistic, nature of DCS, which has been underlined just recently in a quantitative systems pharmacology-based computer modeling of complex humanized brain circuits (Spiros et al., 2014). Another reason might be the possibility of an endocytosis of the receptors, especially considering that weekly rather than daily dosages did show positive effects of DCS on negative symptoms in schizophrenia (Goff et al., 2008). Overall, 2 meta-analyses, balancing the quality discrepancies of the aforementioned studies, find other glutaminergic drugs to be more efficient in schizophrenia than DCS, probably due to its narrow therapeutic window caused by its partial agonistic mechanism (Tuominen et al., 2005; Tsai and Lin, 2010).

In contrast to schizophrenia, an overactive glutamate system is speculated in depression. Therefore, studies in treatment-resistant major depression hypothesize a beneficial antidepressant effect of DCS due to its antagonistic mechanism in higher doses. A first study with 250mg/d DCS as add-on therapy to various stable psychotropic medications failed to show positive results (Heresco-Levy et al., 2006). A further study with a dose of up to 1000mg/d, however, then showed an improvement in depression symptoms (Heresco-Levy et al., 2013), indicating an antagonistic mechanism in doses starting at 500mg/d in humans.

The hypothesis of autism being a hypoglutamatergic disorder was first introduced by Carlsson (1998) and supported by genetic mouse models of the disease (for references, see Urbano et al., 2014). A preliminary study by Posey et al. (2004) showed positive effects of DCS by reducing social withdrawal and increasing social responsiveness. More recently, positive effects of DCS on stereotypies (Urbano et al., 2014) and social deficits (Urbano et al., 2015) were also seen in older adolescents and young adults with autism spectrum disorder. All data so far have to be interpreted with caution, because of several weaknesses of the studies (small sample size, no placebo control, lack of clinical blinding in the study of Posey et al., 2004). Therefore, further studies need to replicate and expand these results.

High expectations have also been raised with regard to a combination of DCS with cognitive behavioral therapy (CBT). The rationale that NMDA receptors are highly involved in learning processes like conditioning and deconditioning (as outlined above) led to several studies in the fields of anxiety and panic disorders as well as dependency. Further supported by animal studies that showed a more rapid extinction of fear with DCS (Walker et al., 2002; Ledgerwood et al., 2003), the hope was that DCS would accelerate the effects of behavioral therapy (especially exposure therapy) and thereby improve therapy compliance and outcome. A huge meta-analysis (Norberg et al., 2008) of data from human and animal studies showed a significant, though moderate, positive effect of DCS on exposure therapy/fear extinction. In anxiety-disordered humans, this was mainly due to a better speed/efficacy of the therapy rather than its better overall outcome, because it produced a faster (and therefore more economic) result (Norberg et al., 2008). This is in line with the results of a recent multi-site study on CBT and DCS in social anxiety disorder (Hofmann et al., 2013). Further promising results have been found, especially for the combination of DCS and CBT, in patients with anorexia nervosa (food exposure therapy) (Steinglass et al., 2007) and obsessive-compulsive and panic disorder as well as with phobias (Davis, 2011). The time-point of drug application (before/after CBT), the frequency of drug administration, and the correct dosages are still the focus of research, mainly because the above-mentioned meta-analysis showed a correlation of these factors with efficacy in augmenting CBT. This seems to be mainly based on the animal data, since a second meta-analysis, this time including only data with humans, could find no such correlations (though it still also underlined the positive effect of DCS in combination with CBT) (Bontempo et al., 2012). From animal models we know that administration of DCS (maximum 4 hours) after a fear extinction experiment shows better results than an application before the experiment (Ledgerwood et al., 2003). It has also been demonstrated that the efficacy of DCS wears off if administered frequently, unless the time interval between applications is long enough (Parnas et al., 2005). Perhaps even just one single application of DCS might be sufficient, as long as it is used to augment de novo CBT (as opposed to repeated CBT) (Gottlieb et al., 2011). For more information on DCS as an augmentation strategy in CBT for anxiety disorders, see 2 detailed recent reviews (Hofmann et al., 2015; Singewald et al., 2015).

In addiction, cue exposure therapy (CET) aims to reduce conditioned reactions to substance cues. This process is believed to be a form of extinction learning rather than a form of “unlearning.” Since NMDA receptors are generally accepted to play a crucial role in extinction learning (eg, fear extinction), it was hypothysed that DCS might enhance CET. Some preliminary data on addiction therapy have shown promising results from using DCS in combination with CET in nicotine (Santa Ana et al., 2009). In cocaine dependency, however, increased craving during the sessions has been reported, maybe due to the glutamatergic effect of DCS (Price et al., 2009). Therefore, the correct timing of drug intake might be crucial to avoid stimulating the patient during cue exposure while also facilitating extinction learning afterwards. Due to weaknesses of the aforementioned studies and the limited number of participants so far (for a detailed review, see Myers and Carlezon, 2012), more studies involving DCS, CET, and addiction are needed, though it should be mentioned that CET itself seems to be still under debate (Conklin and Tiffany, 2002).

### Neurological Diseases

In Alzheimer’s disease, next to the well-established cholinergic deficit, a glutaminergic dysregulation has been found and an NMDA receptor dysfunction has been proven (for details, see Mota et al., 2014). Since positive modulation of NMDA receptors may lead to enhanced memory and learning (through LTP), DCS has been suggested to improve cognitive capacities in Alzheimer’s disease. First studies were disappointing and showed no (Randolph et al., 1994; Fakouhi et al., 1995) or only marginal (Schwartz et al., 1996) effects on cognition. A meta-analysis of 2 large, multi-center parallel group studies of 6-months duration (partially unpublished data) was unable to show that DCS had a positive effect on cognitive outcomes (Jones et al., 2002). Due to its relatively high statistical power, the authors concluded that DCS has no place in the treatment of patients with Alzheimer’s disease. Study designs have to be reconsidered, however. The above-discussed possibility of a wearing-off effect, perhaps due to a downward regulation of the receptor, might be a reason for the weak effect of DCS on cognition in these studies, since DCS had been administered on a daily basis (sometimes twice daily). Only Tsai and colleagues (1999), treating patients with Alzheimer’s disease with DCS once weekly, were able to observe an improvement in cognition.

There has been one study on diseases with spinocerebellar degeneration that investigated the effect of DCS on ataxia symptoms, motivated by the consideration that impaired (mainly cerebellar) glutamatergic projections might be a part of the underlying pathophysiology (Ogawa et al., 2003). The data, though preliminary, are somewhat promising, since they show improvements on ataxia scales, thereby replicating former animal data (Saigoh et al., 1998), but the results still need to be replicated by independent groups with a larger number of subjects.

NMDA receptors are also involved in motor learning through LTP, which has been proven in animal experiments (Hess et al., 1996), and DCS facilitates motor learning in neurophysiological trials (Nitsche et al., 2004). Since motor learning plays a crucial role in neurorehabilitation (eg, after stroke), the rationale of DCS improving outcomes of neurorehabilitative therapies has been drawn. Unfortunately, recent studies failed to show a positive effect of an add-on therapy with DCS to motor/movement therapy (Cherry et al., 2014; Nadeau et al., 2014).

## Conclusions and Future Prospects

Studies with DCS represent an exceptional example of translational scientific work, applying animal data toward the design of human studies. This is not least also due to the fact that DCS had already been approved for human use in other applications. As a well-known drug in tuberculosis therapy, it has been found to be safe, especially in lower dosages, as has again been shown in our review in a new application. The most promising results have been found in combining DCS with CBT in various neuropsychiatric diseases. In accordance, a search for ongoing, registered trials (U.S. National Library of Medicine) reveals mainly studies in this field, examining the effect of DCS on CBT in anxiety disorders, addiction, schizophrenia, and depression. A few exploratory trials also study traumatic brain injury, pain, and dyspnea perception or tinnitus. Nevertheless, newer research is increasingly encountering the limitations of the drug, mainly due to its dose-dependent partially agonistic/antagonistic mechanism (the optimal dosage seems to be crucial, but perhaps inter-individually different), its loss of efficacy on regular application (hypothesized downward-regulation of the receptors), as well as its low specificity with regard to the different subunits of NR2 with probably different pharmacological profiles. Therefore, the step “back to bench” with the development of new (more specific) substances might be worthwhile, while keeping our knowledge of DCS in mind (Monaghan and Larsen, 1997).

## Statement of Interest

None.

## Supplementary Material

supplementary Material
