# Supplementary material for: D-Cycloserine in Neuropsychiatric Diseases: A Systematic Review
Source: Int J Neuropsychopharmacol. 2015 Sep 12;19(4):pyv102. doi: 10.1093/ijnp/pyv102 (PMC4851259; doi:10.1093/ijnp/pyv102)
Supplement: supplementary Material [file pyv102supplement.docx]

**Supplementary Material**

**Table of studies with DCS in humans**

- 1. Studies with DCS in patients with schizophrenia

|  | **Dosing** | **Comedication** | **N DCS** | **N PCB** | **Side Effects** | **Dropout in Verum** | **Dropout in PCB** | **Paraclinics, DCS Plasma Level, Remarks** |
| --- | --- | --- | --- | --- | --- | --- | --- | --- |
| (Goff et al., 1995) | Increasing daily doses of PCP, 5, 15, 50, and 250 mg DCS , each for 2 weeks | Conventional neuroleptics | 10 * | 10 * | None | 1/10 during 50- mg period refusing further assessments (Goff et al. 1996: patient returned and completed study) | None | No change in serum concentration of fluphenazine, or haloperidol (reported in Goff et al. 1996)  DCS: after 12-hour fast at the end of 2-week-trial:   13.6 µM (SD = 15.1) at a dose of 50 mg/d  21.2 µM (SD = 17.8) at a dose of 250 mg/d  (reported in Goff et al. 1996)  *Same subjects in verum and PCB |
| (Rosse et al., 1996) | Daily doses of 10 mg (2 x 5 mg) or 30 mg (2  x  15 mg) DCS or PCB for 4 weeks | Molindone + benztropine mesylate | 9 * | 4 | None | None | None | * 3 (10 mg); 6 (30 mg), |
| (Goff et al., 1996) | Increasing daily doses of PCP, 5, 15, 50, and 250 mg DCS, each for 2 weeks | Clozapine | 10 * | 10 * | Worsening of negative symptoms (interaction with clozapine?) | None | None | No change in serum concentration of clozapine; changes in serum concentration of glutamate and glycine  DCS: after 12-hour fast at the end of 2-week-trial:   8.1 µM (SD = 3.5) at a dose of 50 mg/d  20.7 µM (SD = 16.3) at a dose of 250 mg/d  * Same subjects in verum and PCB |
| (van Berckel et al., 1996) | Increasing daily doses of PCB, 15, 25, 50, 100, and 250 mg, each for 4 days ( = 24 days) | None (only paracetamol and chloralhydrate allowed) | 10 * | 13 * | None | 1/10 during 50-mg period after excerbation of schizophrenic symptoms 1/10 during 50-mg period due to aggressive behavior 1/10 during 100-mg period refusing further assessments | 1/13 during PCB period after cannabis abuse 1/13 during PCB period due to aggressive behavior 1/13 during PCB period not meeting inclusion criterion of moderate symptoms | Ma x imum of 45.8 (±6.9) µmol/L after the 250-mg period Other concentrations shown in a graph  * 10 subjects same in verum and PCB |
| (Heresco-Levy et al., 1998) | Daily doses 50 mg (2 x 25 mg) DCS or PCB for 6 weeks | Atypical antipsychotics | 9 * | 8 * | None | 2/9 in final DCS week due to increased excitement and uncooperativeness | None | No adverse effect on blood chemistry, haematology, liver and kidney function parametersrise of glycine and serine levels, but within normal reference ranges* 8 subjects same in verum and PCB |
| (Goff et al., 1999b) | Daily doses of 50 mg DCS or PCB for 8 weeks | Conventional neuroleptics | 23 | 24 | Besides dropouts, none | 1/23 hospitalized due to worsening psychotic symptoms 1/23 needed medication adjustment due to worsening psychotic symptoms 1/23 citing lack of efficacy 1/23 complaining vague somatic discomfort 3/23 for travel reasons | 1/24 hospitalized due to worsening psychotic symptoms | No change in glycine, glutamate, aspartate, and homovanillic acid DCS concentrations at week 8 correlated significantly with the change from baseline in glutamate and homovanillic acid DCS: after 12-hour fast: highest concentration measured in any subject:  54 μmol/L (drop-out due to clinical worsening) |
| (Goff et al., 1999a) | Daily doses of 50 mg DCS or PCB for 6 weeks | Clozapine | 13 * | 16 * | Moderate worsening of negative symptoms (interaction with clozapine?) | 1/17 during verum period (being the first condition; third week) due to a worsening of psychosis | 4/17 during PCB period (being the first condition) 1/16 during PCB period (being the second condition) | * 13 subjects same in verum and PCB |
| (van Berckel et al., 1999) | Daily doses 100 mg (2 x 50 mg) DCS or PCB for 8 weeks | Antipsychotic and anticholinergic medication no antidepressants | 13 | 13 | Moderate increase in psychotic symptoms and general psychopathology (eg delusions, sexually inappropriate behavior) | 1/13 stating "these pills made me crazy" | None | No change in LH   DCS: stable during the trial (mean = 23.7 µM, SD =  12.2, range mean values between 29.3 µM and 20.3µM) |
| (Heresco-Levy et al., 2002a) | Daily doses of 50 mg or PCB for 6 weeks | Atypical antipsychotics (olanzapine or risperidone), or conventional neuroleptics | 24 * | 24 * | None | 1/24 due to common viral infection 2/24 due to worsening symptoms | 3/24 due to common viral infection  2/24 due to worsening symptoms | No changes in serum chemistry or hematological values  * Same subjects in verum and PCB; due to drop-outs, exact number of really treated subjects is not stated (16 subjects finished the entire trial) |
| (Evins et al., 2002) | Increasing daily doses of PCB, 5, 15, 50, and 250 mg, each for 2 weeks | Risperidone | 10 * | 13 * | None | None | 3/13 during PCB period due to withdrawal of consent | Significant increase of glutamate and serine nonsignificant increase of glycine and aspartate no change in plasma risperidone, or 9-dehydro x yrisperidone  * 10 subjects same in verum and PCB |
| (Duncan et al., 2004) | Daily doses of 50 mg DCS or PCB for 4 weeks | Typical neuroleptics | 10 | 12 | None | None | None | No change in homovanillic acid; no change in plasma level of haloperidol, fluphenazine, or chlorpromazine  DCS: stable during the trial (Day 14: 1.3 ± 0.6 ng/dL; Day 28: 1.2 ± 0.9 ng/dL) |
| (Goff et al., 2005) | Daily doses 50 mg DCS or PCB for 6 months | Conventional antipsychotics | 27 | 28 | High drop-out rate | 2/27 due to adverse events11/27 due to noncompliance or withdrawal of consent | 6/28 due to adverse events10/28 due to noncompliance or withdrawal of consent | No change in glutamate, or glycineDCS: mean DCS concentrations at week 8 in responders (n = 7) was 169 μM compared with 186 μM in nonresponders (n = 8) |
| (Yurgelun-Todd et al., 2005) | Daily doses of 50 mg DCS or PCB for 8 weeks | Conventional neuroleptics (eg, haloperidol, fluphenazine) | 6 | 6 | No information | None | None | n/a |
| (Buchanan et al., 2007) | Daily doses of PCB, glycine, or DCS (7 days 25 mg/d, from day 8 50 mg/d) for 16 weeks | Partially concomitant medication (eg, second-generation antipsychotics) | 56 | 55 | No significant DCS/PCB side effect differences (all *P* > .12) | 3/56 deterioration of psychiatric condition 2/56 side effects of the study medication 4/56 subject request 1/56 due to "other" | 2/55 deterioration of psychiatric condition 2/55 noncompliance 2/55 side effects of the study medication 3/55 unrelated medical reason 1/55 due to "other" | Median increases in glycine and serine were near zero  DCS: median (interquartile range) attained plasma DCS levels for observed cases were 0.80 mg/dL (range = 0.60–1.20) at weeks 4 and 8 and 0.75 mg/dL (range = 0.50–1.00) at week 16. |
| (Goff et al., 2008) | Weekly doses of 50 mg DCS or PCB for 8 weeks | Antipsychotic medication except clozapine | 19 | 19 | DCS: bronchitis, depression, fatigue, vomiting (each in 1/19)  headache, URI (each in 2/19) PCB: anxiety, chest pain, fracture, vertigo (each in 1/19) depression, diarrhea, heart burn, URI (each in 2/19) | 1/19 due to worsening of psychosis after discontinuing antipsychotic medication 1/19 medication noncompliance 1/19 due to elective surgery | 2/19 lost to follow-up | n/a |
| (Gottlieb et al., 2011) | Single dose of 50 mg DCS or PCB | Partially concomitant medication (except for clozapine) | 20 * | 20 * | None | None | None | * Same subjects in verum and PCB |

- 1. Studies with DCS in patients with an x iety disorders

|  | **Dosing** | **Comedication** | **N DCS** | **N PCB** | **Side Effects** | **Dropout in Verum** | **Dropout in PCB** | **Paraclinics, DCS Plasma Level, Remarks** |
| --- | --- | --- | --- | --- | --- | --- | --- | --- |
| (Heresco-Levy et al., 2002b) | Daily doses of 50 mg (2 x 25 mg) DCS or PCB for 4 weeks | Partially concomitant medication | 11 * | 11 * | None | 4/11 due to medical problems not related to the experimental medication or study procedures | | No adverse effects on blood chemistry, haematology, liver and kidney function parameters  * Same subjects in verum and PCB |
| (Ressler et al., 2004) | 2 Single doses of PCB, 50 or 500 mg DCS separated by 1-2 weeks | No information | 17 * | 10 | None | 4/17 lost to follow-up after 3 months | 2/10 lost to follow-up after 3 months | * 8 (50 mg); 9 (500 mg) |
| (Hofmann et al., 2006) | 4 Single doses of 50 mg DCS or PCB separated by 1 week | Partially concomitant medication (benzo-diazepine, antid-epressants, ß-blocker, stimulants) | 15 | 17 | 1/15 vivid nightmares 1/15 euphoric mood and increased energy * | 3/15 due to withdrawal or protocol violation 2/15 lost to 1-month-follow-up | 2/17 due to withdrawal 2/17 lost to 1-month-follow-up | * Subjects could not distinguish PCB and DCS |
| (Guastella et al., 2007b) | Single dose of 50 mg, 500 mg or PCB | No information | 51* | 49 | No information | None | None | * 43 (50 mg); 8 (500 mg) |
| (Guastella et al., 2007a) | Single dose of 50 mg, 500 mg or PCB | No information | 122 * | 116 | No information | 24 participants were excluded due to e x perimenter error, data corruption, equipment malfunction, drop out or failure to follow | | * 100 (50 mg); 22 (500 mg) |
| (Storch et al., 2007) | Weekly doses of 250 mg DCS or PCB for 12 weeks | Partially concomitant medication (eg, SSRI) | 12 | 12 | DCS: increased anxiety (1), drowsiness (1) and dry mouth (1) PCB: drowsiness (2) and restlessness (1) | 1/12 lost to follow-up (unable to be reached) | 1/12 lost to follow-up (unable to be reached) | n/a |
| (Kushner et al., 2007) | 10 Single doses of 125 mg DCS or PCB | Partially concomitant medication | 15 | 17 | DCS: mild gastrointestinal distress (1), dizziness (1), fatigue (1), and anxiety (1)  PCB: “jittery feelings” (1), dissociation (1), and dry lips (1) | 1/15 no further information | 6/15 no further information | n/a |
| (Guastella et al., 2008) | Weekly doses of 50 mg DCS or PCB for 4 weeks | Partially concomitant medication | 28 | 28 | None | 1/28 (no information on reasons) | 5/28 (no information on reasons) | n/a |
| (Wilhelm et al., 2008) + (Chasson et al., 2010) | Twice weekly doses of 100 mg DCS or PCB for 5 weeks | Partially concomitant medication | 14 | 15 | None | 4/14 before mid-treatment (no information on reasons) | 3/15 before mid-treatment (no information on reasons) | n/a |
|  |  |  |  |  |  | 1/29 after mid-treatment (no information on reasons) | |  |
| (Otto et al., 2010) | 3 Single doses of 50 mg DCS or PCB | Partially concomitant medication | 15 | 16 | None | 1/15 discontinued intervention 1/15 subject lost to follow-up | 1/15 discontinued intervention 1/15 subject lost to follow-up | n/a |
| (Aupperle et al., 2009) | Single dose of 100 mg DCS or PCB | No psychotropics | 23 | 23 | None | 1/54 due to claustrophobia (not counted in numbers of verum/PCB) 7/54 due to scanner or motion artifacts (not counted in numbers of verum/PCB) | | n/a |
| (Behar et al., 2010) | Single dose of 50 mg DCS or PCB | No psychotropic medications or medications contraindicated with DCS | 22 * | 22 * | No information | 3/44 lost to follow-up | | * 44 patients were randamized, exact allocation not stated though, therefore speculated number |
| (Storch et al., 2010) | Single doses of DCS (children weighing 25 till 45 kg: 25 mg; children weighing 46 till 90 kg: 50 mg) or PCB in 7 sessions at least 5 days apart | Partially concomitant medication (eg psychotropic medication) | 15 | 15 | None | None | None | Complete blood counts, liver function tests, electrolytes, blood urea nitrogen, and creatinine were all normal at enrollment and after treatment with DCS. |
| (Siegmund et al., 2011) | 8 Single doses of 50 mg DCS or PCB in a 4-week time span | Partially concomitant psychopharmacological medication (n = 15)* | 22 | 22 | None | 2/22 decline further participation in exposure treatment | 1/22 due to family problems 1/22 decline of further psychotherapy 1/22 proposed "side effects" of study medication | * = Citalopram, paroxetine, fluoxetine, doxepine, mirtazapin, opipramol, trimipramine, medazepam, diazepam, pregabalin |
| (de Kleine et al., 2012) | Weekly doses of 50 mg DCS or PCB for ma x . 10 weeks | Partially concomitant medication | 33 | 34 | DCS: increased anxiety (2), headache (3), dizziness (3), drowsiness (2).  PCB: depressed mood (3), headache (2), drowsiness (2), bowel pain (2)* | 9/33 One patient was admitted to the hospital, presenting with loss of motor function, disorientation, and severe headache. A suspected relationship with DCS was "ruled out". | 13/34 One patient reporting persistent dizziness was excluded from the study on the advice of an independent physician. | * Subjects could not distinguish PCB and DCS |
| (Litz et al., 2012) | Weekly doses of 50 mg DCS or PCB for 4 weeks | Partially concomitant medication | 13 | 13 | No serious side effects | 4/13 withdrew 3/13 lost to follow-up | 2/13 unable to contact 1/13 withdrew 3/13 lost to follow-up | n/a |
| (Gutner et al., 2012) | Single dose of 50 mg DCS or PCB | No current use of psychiatric or antibiotic medication | 24 | 24 | No adverse events reported Adverse effects in DCS and PCB were not significantly different | None | 3/24 lost to follow-up | n/a |
| (Nave et al., 2012) | Single dose of 50 mg DCS or PCB | Partially concomitant medication | 10 | 10 | No information | None | None | n/a |
| (Tart et al., 2013) | Weekly doses of 50 mg DCS or PCB one week apart for 2 weeks | No use of psychotropic medication | 15 | 14 | No information | 3/15 lost to follow-up | 1/14 no information on reasons 3/14 lost to post-treatment sessions/follow-up | n/a |
| (Hofmann et al., 2013) | 5 Single doses of 50 mg DCS or PCB 1 week apart | No psychotropic medication (eg, antidepressants, anxiolytics, betablockers) | 87 | 82 |  | 8/87 due to time reasons or associated with exposure therapy 1/87 hospitalized |  |  |
| (Rodebaugh et al., 2013) | Single dose of 250 mg DCS or PCB | Partially concomitant medication (antidepressants and benzodiazepines) | 18 | 16 | "Tingling head" (during PCB) | 2/18 lost to follow-up 2/18 withdrew | 2/18 lost to follow-up 2/18 withdrew | n/a |
| (Farrell et al., 2013) | Weekly doses of 25 mg/50 mg (dependent on child weight: <45 kg = 25 mg, and >46 kg = 50 mg) DCS or PCB for 5 weeks | Partially concomitant medication (76% serotonergic reuptake inhibitors) | 9 | 8 | No "adverse events" | 1/9 no information on reasons | 2/8 no information on reasons | Laboratory tests (eg, complete blood count, metabolic panel) remaining within normal limits |
| (Rothbaum et al., 2014) | Weekly doses of 50 mg DCS or PCB (or 0.25 mg alprazolam*) for 5 weeks | Partially concomitant medication (but no glucocorticoids, benzodiazepines, chronically used opioids) | 53 | 53 | No information | No significant differences in dropout rate across conditions | n/a | * Additional 50 patients randomized to alprazolam group |
| (Difede et al., 2014) | Weekly doses of 100 mg DCS or PCB for 10 weeks | Partially concomitant medication (DCS: 8 antidepressants, 4 other psychiatric medications, 4 both; PCB: 4 antidepressants, 5 other psychiatric medications, 4 both ) | 13 | 12 | None | None | 3/12 | n/a |
| (Scheeringa and Weems, 2014) | 7 Single doses of 50 mg DCS or PCB | Partially concomitant medication | 29 | 28 | DCS: 23% reported adverse events (drowsiness (6%), irritability (6%), dizziness (3%), headache (3%), and dry mouth (3%)) PCB: 26% reported adverse events (drowsiness (13%), dizziness (10%), lips burning (3%), increased appetite (3%), stomach pain (3%), and feeling as if ‘‘things going too fast’’ (3%) | 6/29 2/29 lost to follow-up | 4/28 6/28 lost to follow-up | n/a |
| (Attari et al., 2014) | Daily doses of 50 mg (2 x 25 mg) DCS or PCB for 4 weeks | Various psycho-pharmacologic treatments (all patients) | 62 * | 63* | Reported, but not statistically remarkabel side-effects were mild head ache and mild nausea | None | 1/63 during first first period due to psychotic mania | Clinical examination and paraclinical tests (electrocardiography, blood cell count and chemistry, thyroid, liver and renal function tests) did not reveal considerable changes  *Same subjects in verum and PCB |
| (Matai x -Cols et al., 2014) | Weekly doses of with 50 mg DCS or PCB for 10 weeks | Partially concomitant medication (DCS: 4 SSRI; PCB: 3 SSRI, 1 risperidone) | 13 | 13 | No adverse drug reactions to DCS or PCB | 1/13 lost to follow-up | 1/13 refused to receive medication after session 2 | n/a |

- 1. Studies with DCS in patients with addiction

|  | **Dosing** | **Comedication** | **N DCS** | **N PCB** | **Side Effects** | **Dropout in Verum** | **Dropout in PCB** | **Paraclinics, DCS Plasma Level, Remarks** |
| --- | --- | --- | --- | --- | --- | --- | --- | --- |
| (Oliveto et al., 2003) | Single doses of 0, 125, 250 or 500 mg/70 kg DCS alone or with naloxone | Methadone or levomethadyl; naloxone | 6 * | 6 * | None (subjects could not discriminate PCB) | None | 1/7 due to child care issues (before any treatment with DCS) | Significant dose-related increases in systolic blood pressure (interaction with methadone?)  * Same subjects in verum and PCB |
| (Santa Ana et al., 2009) | 2 Single dose of 50 mg DCS or PCB | No psychoactive medication or medication for smoking cessation | 12 | 13 | None | 4/12 no information on reasons | 3/13 no information on reasons | n/a |
| (Price et al., 2009) | 2 Single dose of 50 mg DCS or PCB one day apart | None, but cocaine dependency | 5 | 5 | No information | None | None | n/a |
| (Krystal et al., 2011) | 4 Sessions separated by 3-7 days with PCB-DCS + glycine; DCS + PCB-glycine; DCS + glycine; or PCB-DCS + PCB glycine. (DCS = 1000 mg, glycine = 0.3g/kg) | Glycine | 49 * | 49 * | No information | 7/49 (no information on reasons) * | | Plasma glycine levels increased to a similar extent when glycine was administered by itself or in combination with DCS.  DCS: DCS and D/L-serine plasma levels were significantly lower on days when glycine, rather than PCB, was infused.  * 49 subjects compleated at least one session, exact details on subgroups not given |
| (Nesic et al., 2011) | Single dose of 50 mg DCS or PCB | None, nicotine | 24 | 24 | Feeling "stimulated" and  increased diastolic blood pressure (when in combination with nicotine) | None | None | n/a |
| (Watson et al., 2011) | 2 Single doses of 250 mg DCS or PCB at least 1 week apart | Partially concomitant medication | 8 | 8 | None | 1/8 due to new address unknown | 1/8 due to new address unknown | n/a |
| (Kamboj et al., 2011) | 2 Single doses of 125 mg or PCB at least 2 days apart | No information | 19 | 17 | Mild subjective effects (increased euphoria/contentedness) | None | None | n/a |
| (Hofmann et al., 2012) | 3 Single doses of 50 mg DCS or PCB at least 3 days apart | No psychotropic medication | 10 | 10 | None | None | None | n/a |
| (Kalechstein et al., 2012) | Single dose of 50 mg DCS or PCB | None, but cocaine dependency | 13 | 14 | No information | None | None | n/a |
| (Kamboj et al., 2012) | 2 Single doses of 125 mg DCS or PCB at least 2 days apart | No information | 16 | 16 | None * | None | None | * Subjects could not distinguish PCB and verum |
| (Kennedy et al., 2012) | Weekly doses of 50 mg DCS or PCB for 4 weeks | No use of psychotropic medication, but cocaine dependency | 15 | 13 | No information | None | None | n/a |
| (Price et al., 2013) | 2-3 Single dose of 50 mg DCS or PCB 2 days apart | None, but cocaine dependency | 22 | 10 | No information | None | None | n/a |
| (Yoon et al., 2013) | Weekly doses of 50 mg DCS or PCB for 4 weeks | No use of any psychoactive medication, but cocaine dependency | 21 | 22 | No serious adverse events AEs in DCS and PCB were not significantly different | 2/21 for personal reasons 1/22 discharged after missing 3 consecutive visits | 6/22 for personal reasons | No differences in cardiovascular measures (heart rate, blood pressure) |
| (Prisciandaro et al., 2013a) | 2 Single doses of 50 mg DCS or PCB approximately 48 hours apart | None | 15 | 15 | No information | None | None | Subgroup of a large-scale, yet unpublished study |
| (Prisciandaro et al., 2013b) | 2 Single doses of 50 mg or PCB approximately 48 hours apart | None | 10 | 15 | No information | None | None | Subgroup of a large-scale, yet unpublished study |

- 1. Studies with DCS in patients with dementia

|  | **Dosing** | **Comedication** | **N DCS** | **N PCB** | **Side Effects** | **Dropout in Verum** | **Dropout in PCB** | **Paraclinics, DCS Plasma Level, Remarks** |
| --- | --- | --- | --- | --- | --- | --- | --- | --- |
| (Randolph et al., 1994) | Daily doses of 25, 50, 100, 175, 300 and 500 mg (increasing weekly) | No information given | 12 * | 12 * | 1 subjects transient agitated-confusional state during 500 mg period (plasma DCS level: 54.4 µg/ml) | None | None | DCS: 2 hours after dosing:   3.7 µg/mL at a dose of 25 mg/d   27.0 µg/mL at a dose of 500 mg/d  (other concentrations shown in graph)  * Same subjects in verum and PCB |
| (Fakouhi et al., 1995) | Daily doses of 10 mg (2 x 5 mg), 30 mg (2 x 15 mg), 100 mg (2 x 50 mg) DCS or PCB for 26 weeks | Partially concomitant medication | 102 (10 mg) 102 (30 mg) 99 (100 mg) | 107 | Comparable among groups (upper respiratory tract infections, headache, accidental injury, agitation, arthrits, and confusion) | 35/102 in 10 mg-group 36/102 in 30 mg-group 37/99 in 100 mg-group  (no information on reasons) | 29/99 (no information on reasons) | No clinically significant laboratory abnormalities were observed |
| (Schwartz et al., 1996) | Daily doses of 10 mg (2 x 5 mg), 30 mg (2 x 15 mg), 100 mg (2 x 50 mg) DCS or PCB for 10 weeks | No CNS-affecting medication | 71 * | 20 | No information given | 17/108 (no further information on reasons or treatment group) | | * 22 (5 mg), 25 (15 mg), 24 (50 mg) |
| (Tsai et al., 1998) | Daily doses of 15 mg DCS or PCB for 4 weeks | No information given | 10 * | 10 * | None | None | None | * Same subjects in verum and PCB |
| (Tsai et al., 1999) | Daily doses of 50 mg, 150 mg DCS or PCB for 4 weeks | No information given | 17 * | 17 * | None | None | None | * Same subjects in verum and PCB |

- 1. Studies with DCS in patients with other pathological conditions

|  | **Dosing** | **Comedication** | **N DCS** | **N PCB** | **Side Effects** | **Dropout in Verum** | **Dropout in PCB** | **Paraclinics, DCS Plasma Level, Remarks** |
| --- | --- | --- | --- | --- | --- | --- | --- | --- |
| (Ogawa et al., 2003) | Daily doses of 50 mg (2 x 25 mg) for 2 weeks with 2-week PCB lead-in | No information | 15 * | 15 * | None | None | None | No laboratory abnormalities (hematology, biochemistry and urinalysis)  * Same subjects in verum and PCB |
| (Posey et al., 2004) | Daily doses of approximately 0.7, 1.4, and 2.8 mg DCS/kg/d or PCP increasing each for 2 weeks | None | 10 * | 12 * | 1/10 Motoric tic during 2.8 mg/kg/d period  1/10 increased echolalia during 3.0 mg/kg/d | None | 1/12 due to worsening stereotypic behavior  1/12 due to non-compliance | No significant change in laboratory values and physical examination  * 10 subjects same in verum and PCB |
| (Heresco-Levy et al., 2006) | Daily doses of 250 mg DCS or PCB for 6 weeks | Antidepressant medication (eg, clonazepam, mianserin) | 19 | 20 | None | 1/19 due to symptom exacerbation 1/19 due to medical reason not related to treatment | 4/20 due to symptom exacerbation 1/20 due to medical reasons | No laboratory side effects were registered |
| (Steinglass et al., 2007) | 4 Single doses of 50 mg DCS or PCB (twice weekly on nonconsecutive days) | No psychotropic medications | 6 * | 6 * | None | 2/13 after first and second session (no Information on reasons) | | No medication-related laboratory abnormalities were noted  * 13 subjects were randomized, no exact information on actual allocation, therefore speculated numbers |
| (Heresco-Levy et al., 2013) | Daily doses of 250 mg DCS for 3 days, 500 mg DCS for 18 days, 750 mg DCS for 1 week and 1000 mg DCS or PCB for 2 weeks | One or more antidepressant drugs | 13 | 13 | Side-effects were mild and did not differ significantly in the two treatment groups No neurological side-effects | 1/13 due to hearing discomfort 1/13 due to noncompliance 1/13 due to tiredness | 1/13 due to chest pain | n/a |
| (Chan et al., 2013) | Daily doses of 100 mg DCS for 20 days | No information | 5 | None | None | None | n/a | n/a |
| (Urbano et al., 2014) | Daily or weekly doses of 50 mg DCS for 8 weeks | Partially concomitant medication (13 serotonin enhancing drugs, 9 stimulants *, 3 risperidone or aripiprazole, 1 clonidine, 1 oxcarbazepine) | 20 | None | Only transient spontaneously recorded adverse effects | None | n/a | More detailed data yet unpublished  * Methylphenidate, atomoxetine, dextro-/amphetamine, |
| (Nadeau et al., 2014) | Daily doses of 50 mg or PCB for 10 consecutive days or single doses of 50 mg or PCB on 3 days per week in 10 consecutive weeks | No information | 12 | 12 | No adverse events associated with DCS, 1/12 small skin tear and a sore elbow | 1/12 lost to follow-up (moved out of state) | 1/12 during treatment due to recurrent stroke | n/a |
| (Cherry et al., 2014) | Single dose of 250 mg DCS or PCB | No information | 29 | 32 | No information | None | None | n/a |

- 1. Studies with DCS in healthy subjects

|  | **Dosing** | **Co-medication** | **N DCS** | **N PCB** | **Side Effects** | **Dropout in Verum** | **dropout in PCB** | **Paraclinics, DCS plasma level, remarks** |
| --- | --- | --- | --- | --- | --- | --- | --- | --- |
| (van Berckel et al., 1997) | Single dose of 15, 50, 150 mg DCS or PCB, each at least 1 week apart | None | 16 * | 16 * | 6/16 mild headache (1 during PCB, 2 during 15 mg, 1 during 50 mg, 2 during 150 mg) 1/16 blurred vision (during 15 mg) 1/16 nausea (during PCB) | None | None | No change in LH, cortisol, or prolactin No effect on blood pressure, heart rate, or temperature  DCS: concentrations shown in a graph  * Same subjects in verum and PCB |
| (van Berckel et al., 1998) | Single dose of 500 mg DCS or PCB | None | 20 * | 20 * | 3/20 mild insignificant headache (during verum) 1/20 vision problems (during PCB) | None | None | DCS induced a significant rise in LH plasma levels, starting at 60 min after treatment no change in cortisol and homovanillic acid no effect on blood pressure, heart rate and temperature  DCS: At 60 minutes after administration, the mean plasma level of DjCS was 113 µM (range 5-209 µM), decreasing slightly after 240 min to 100 µM (range 61-139 µM)  * Same subjects in verum and PCB |
| (D'Souza et al., 2000) | 50 mg DCS + PCB glycine, or 3 other conditions with PCB DCS (randomized counterbalanced order with minimum 3-day interval) | None | 26 * | 26 * | 6/26 post-LP headache | 6/26 did not complete all 4 sessions 1/26 due to late admittance of history of alcohol abuse | | No change in plasma or cerebrospinal fluid amino acids (glycine, serine etc.),  No change in plasma prolactin, cortisol, growth hormone, or homovanillic acid No change in cerebrospinal fluid 5HIAA, methoxyhydroxypheylglycol, or homovanillic acid  * Same subjects in verum and PCB, but due to drop-outs, e x act number of really treated subjects is not stated |
| (Nitsche et al., 2004) | Three single doses of 100 mg DCS or PCB | None | 12 | 12 | None | None | None | * Same subjects in verum and PCB |
| (Bailey et al., 2007) | Single dose of 50 mg DCS or PCB | CO2-inhalation | 24 | 24 | Dizziness, headache and feelings of anxiety and breathlessness (probably related to CO2-inhalation) | None | None | DCS showed no effect on blood pressure or heart rate |
| (Britton et al., 2007) | Single dose of 500 mg DCS or PCB | None | 7 | 7 | None | None | None | n/a |
| (Teo et al., 2007) | Single dose of 100 mg DCS or PCB | None | 6 * | 6 * | None  ** | None | None | * Same subjects in verum and PCB  ** Subjects could not distinguish PCB and verum |
| (Kuo et al., 2008) | Single dose of 100 mg DCS or PCB | None | 80 * | 80 * | No information | None | None | * Same subjects in verum and PCB |
| (Otto et al., 2009) | Weekly doses of 50 mg DCS or PCB | None | 18 | 17 | No information | 1/18 (no information on reasons) | 1/17 (no information on reasons) | n/a |
| (Kalisch et al., 2009) | Single dose of 500 mg DCS or PCB | None | 16 | 16 | 6/15 headache 3/16 tiredness 3/16 dizziness 2/16 nausea 1/16 feeling hot/sweaty | 1/16 headache | none | n/a |
| (Onur et al., 2010) | Single dose of 250 mg DCS or PCB | None | 20 | 20 | No information | None | None | n/a |
| (Kuriyama et al., 2011a) | Single dose of 100 mg DCS, 400 mg VPA + 100 mg DCS, 400 mg VPA or PCB | Valproic acid (as study medication) | 31 * | 14 | 2 subjects in each treatment group reported slight drowsiness | None | None | * 16 subjects received DCS, 15 subjects received DCS + VPA |
| (Kuriyama et al., 2011b) | Single dose of 100 mg DCS, 400 mg VPA + 100 mg DCS, 400 mg VPA or PCB | VPA (as study medication) | 31* | 15 | 2 subjects in each treatment group reported drowsiness | None | 1/15 due to a "bad cold" | * 16 subjects received DCS, 15 subjects received DCS + VPA |
| (Kuriyama et al., 2011c) | Single dose of 100 mg DCS or PCB | None, but cocaine dependency | 45 | 43 | 2–3 subjects in each treatment group reported drowsiness subjects could not distinguish PCB and verum | None | None | n/a |
| (Chaieb et al., 2012) | Two single dose of 100 mg DCS, 0.025 mg pergolide + 10 mg domperidone or PCB | None | 8 * | 8 * | None | None | None | * Same subjects for all conditions |
| (Klumpers et al., 2012) | Single dose of 250 mg DCS, 10 mg delta-9- tetrahydrocannabinol or PCB | None | 18 | 18 | None | None | None | n/a |
| (Kuriyama et al., 2013) | Single dose of 100 mg DCS, 400 mg VPA or PCB | None | 28 | 29 | DCS "did not produce any specific subjective side effects in the subjects" DCS "did not produce any severe objective psychomotor side effercts in subjects" | None | None | n/a |
| (Feld et al., 2013) | Single dose of 175 mg DCS or PCB 2 separated by 2 weeks | None | 30 * | 30 * | None ** | None | None | Levels of cortisol and ACTH did not differ between treatment conditions   * Same subjects in verum and PCB  ** Subjects could not distinguish PCB and verum |
| (Scholl et al., 2014) | Single dose of 250 mg DCS or PCB | No CNS-active medication | 20 | 27 | No information | None | None | n/a |

**REFERENCES**

Attari A, Rajabi F, Maracy MR (2014) D-cycloserine for treatment of numbing and avoidance in chronic post traumatic stress disorder: A randomized, double blind, clinical trial. J Res Med Sci 19:592–598.

Aupperle RL, Hale LR, Chambers RJ, Cain SE, Barth FX , Sharp SC, Denney DR, Savage CR (2009) An fMRI study examining effects of acute D-cycloserine during symptom provocation in spider phobia. CNS spectrums 14:556–571.

Bailey JE, Papadopoulos A, Lingford-Hughes A, Nutt DJ (2007) D-Cycloserine and performance under different states of anxiety in healthy volunteers. Psychopharmacology (Berl) 193:579–585.

Behar E, McHugh RK, Peckham A, Otto MW (2010) D-cycloserine for the augmentation of an attentional training intervention for trait an x iety. J Anxiety Disord 24:440–445.

Britton JC, Gold AL, Feczko EJ, Rauch SL, Williams D, Wright CI (2007) D-cycloserine inhibits amygdala responses during repeated presentations of faces. CNS spectrums 12:600–605.

Buchanan RW, Javitt DC, Marder SR, Schooler NR, Gold JM, McMahon RP, Heresco-Levy U, Carpenter WT (2007) The Cognitive and Negative Symptoms in Schizophrenia Trial (CONSIST): the efficacy of glutamatergic agents for negative symptoms and cognitive impairments. Am J Psychiatry 164:1593–1602.

Chaieb L, Antal A, Terney D, Paulus W (2012) Pharmacological modulation of the short-lasting effects of antagonistic direct current-stimulation over the human motor cortex . Front Psychiatry 3:67.

Chan HN, Alonzo A, Martin DM, Mitchell PB, Sachdev P, Loo CK (2013) Augmenting transcranial direct current stimulation with (D)-cycloserine for depression: a pilot study. J ECT 29:196-200.

Chasson GS, Buhlmann U, Tolin DF, Rao SR, Reese HE, Rowley T, Welsh KS, Wilhelm S (2010) Need for speed: evaluating slopes of OCD recovery in behavior therapy enhanced with d-cycloserine. Behav Res Ther 48:675–679.

Cherry KM, Lenze EJ, Lang CE (2014) Combining d-cycloserine with motor training does not result in improved general motor learning in neurologically intact people or in people with stroke. J Neurophysiol 111:2516–2524.

D'Souza DC, Gil R, Cassello K, Morrissey K, Abi-Saab D, White J, Sturwold R, Bennett A, Karper LP, Zuzarte E, Charney DS, Krystal JH (2000) IV glycine and oral D-cycloserine effects on plasma and CSF amino acids in healthy humans. Biol Psychiatry 47:450–462.

de Kleine RA, Hendriks GJ, Kusters WJ, Broekman TG, van Minnen A (2012) A randomized placebo-controlled trial of D-cycloserine to enhance exposure therapy for posttraumatic stress disorder. Biol Psychiatry 71:962–968.

Difede J, Cukor J, Wyka K, Olden M, Hoffman H, Lee FS, Altemus M (2014) D-cycloserine augmentation of exposure therapy for post-traumatic stress disorder: a pilot randomized clinical trial. Neuropsychopharmacology 39:1052–1058.

Duncan EJ, Szilagyi S, Schwartz MP, Bugarski-Kirola D, Kunzova A, Negi S, Stephanides M, Efferen TR, Angrist B, Peselow E, Corwin J, Gonzenbach S, Rotrosen JP (2004) Effects of D-cycloserine on negative symptoms in schizophrenia. Schizophr Res 71:239–248.

Evins AE, Amico E, Posever TA, Toker R, Goff DC (2002) D-Cycloserine added to risperidone in patients with primary negative symptoms of schizophrenia. Schizophr Res 56:19–23.

Fakouhi TD, Jhee SS, Sramek JJ, Benes C, Schwartz P, Hantsburger G, Herting R, Swabb EA, Cutler NR (1995) Evaluation of cycloserine in the treatment of Alzheimer's disease. J Geriatr Psychiatry Neurol 8:226–230.

Farrell LJ, Waters AM, Boschen MJ, Hattingh L, McConnell H, Milliner EL, Collings N, Zimmer-Gembeck M, Shelton D, Ollendick TH, Testa C, Storch EA (2013) Difficult-to-treat pediatric obsessive-compulsive disorder: feasibility and preliminary results of a randomized pilot trial of D-cycloserine-augmented behavior therapy. Depress Anxiety 30:723–731.

Feld GB, Lange T, Gais S, Born J (2013) Sleep-dependent declarative memory consolidation--unaffected after blocking NMDA or AMPA receptors but enhanced by NMDA coagonist D-cycloserine. Neuropsychopharmacology 38:2688–2697.

Goff DC, Tsai G, Manoach DS, Coyle JT (1995) Dose-finding trial of D-cycloserine added to neuroleptics for negative symptoms in schizophrenia. Am J Psychiatry 152:1213–1215.

Goff DC, Henderson DC, Evins AE, Amico E (1999a) A placebo-controlled crossover trial of D-cycloserine added to clozapine in patients with schizophrenia. Biol Psychiatry 45:512–514.

Goff DC, Tsai G, Manoach DS, Flood J, Darby DG, Coyle JT (1996) D-cycloserine added to clozapine for patients with schizophrenia. Am J Psychiatry 153:1628–1630.

Goff DC, Tsai G, Levitt J, Amico E, Manoach D, Schoenfeld DA, Hayden DL, McCarley R, Coyle JT (1999b) A placebo-controlled trial of D-cycloserine added to conventional neuroleptics in patients with schizophrenia. Arch Gen Psychiatry 56:21–27.

Goff DC, Cather C, Gottlieb JD, Evins AE, Walsh J, Raeke L, Otto MW, Schoenfeld D, Green MF (2008) Once-weekly D-cycloserine effects on negative symptoms and cognition in schizophrenia: an exploratory study. Schizophr Res 106:320–327.

Goff DC, Herz L, Posever T, Shih V, Tsai G, Henderson DC, Freudenreich O, Evins AE, Yovel I, Zhang H, Schoenfeld D (2005) A si x -month, placebo-controlled trial of D-cycloserine co-administered with conventional antipsychotics in schizophrenia patients. Psychopharmacology (Berl) 179:144–150.

Gottlieb JD, Cather C, Shanahan M, Creedon T, Macklin EA, Goff DC (2011) D-cycloserine facilitation of cognitive behavioral therapy for delusions in schizophrenia. Schizophr Res 131:69–74.

Guastella AJ, Lovibond PF, Dadds MR, Mitchell P, Richardson R (2007a) A randomized controlled trial of the effect of D-cycloserine on e x tinction and fear conditioning in humans. Behav Res Ther 45:663–672.

Guastella AJ, Dadds MR, Lovibond PF, Mitchell P, Richardson R (2007b) A randomized controlled trial of the effect of D-cycloserine on e x posure therapy for spider fear. J Psychiatr Res 41:466–471.

Guastella AJ, Richardson R, Lovibond PF, Rapee RM, Gaston JE, Mitchell P, Dadds MR (2008) A randomized controlled trial of D-cycloserine enhancement of exposure therapy for social anxiety disorder. Biol Psychiatry 63:544–549.

Gutner CA, Weinberger J, Hofmann SG (2012) The effect of D-cycloserine on subliminal cue exposure in spider fearful individuals. Cogn Behav Ther 41:335–344.

Heresco-Levy U, Javitt DC, Ermilov M, Silipo G, Shimoni J (1998) Double-blind, placebo-controlled, crossover trial of D-cycloserine adjuvant therapy for treatment-resistant schizophrenia. Int J Neuropsychopharmacol 1:131–135.

Heresco-Levy U, Ermilov M, Shimoni J, Shapira B, Silipo G, Javitt DC (2002a) Placebo-controlled trial of D-cycloserine added to conventional neuroleptics, olanzapine, or risperidone in schizophrenia. Am J Psychiatry 159:480–482.

Heresco-Levy U, Kremer I, Javitt DC, Goichman R, Reshef A, Blanaru M, Cohen T (2002b) Pilot-controlled trial of D-cycloserine for the treatment of post-traumatic stress disorder. Int J Neuropsychopharmacol 5:301–307.

Heresco-Levy U, Javitt DC, Gelfin Y, Gorelik E, Bar M, Blanaru M, Kremer I (2006) Controlled trial of D-cycloserine adjuvant therapy for treatment-resistant major depressive disorder. J Affect Disord 93:239–243.

Heresco-Levy U, Gelfin G, Bloch B, Levin R, Edelman S, Javitt DC, Kremer I (2013) A randomized add-on trial of high-dose D-cycloserine for treatment-resistant depression. Int J Neuropsychopharmacol 16:501–506.

Hofmann SG, Huweler R, MacKillop J, Kantak KM (2012) Effects of D-cycloserine on craving to alcohol cues in problem drinkers: preliminary findings. Am J Drug Alcohol Abuse 38:101–107.

Hofmann SG, Meuret AE, Smits JA, Simon NM, Pollack MH, Eisenmenger K, Shiekh M, Otto MW (2006) Augmentation of e x posure therapy with D-cycloserine for social an x iety disorder. Arch Gen Psychiatry 63:298–304.

Hofmann SG, Smits JA, Rosenfield D, Simon N, Otto MW, Meuret AE, Marques L, Fang A, Tart C, Pollack MH (2013) D-Cycloserine as an augmentation strategy with cognitive-behavioral therapy for social anxiety disorder. Am J Psychiatry 170:751–758.

Kalechstein AD, Yoon JH, Mahoney JJ III Newton TF, Chang L, De La Garza R II (2012) d-Cycloserine administration does not affect neurocognition in concurrent cocaine- and nicotine-dependent volunteers. Pharmacol Biochem Behav 103:403–407.

Kalisch R, Holt B, Petrovic P, De Martino B, Kloppel S, Buchel C, Dolan RJ (2009) The NMDA agonist D-cycloserine facilitates fear memory consolidation in humans. Cereb Cortex  19:187–196.

Kamboj SK, Joye A, Das RK, Gibson AJ, Morgan CJ, Curran HV (2012) Cue exposure and response prevention with heavy smokers: a laboratory-based randomised placebo-controlled trial examining the effects of D-cycloserine on cue reactivity and attentional bias. Psychopharmacology (Berl) 221:273–284.

Kamboj SK, Massey-Chase R, Rodney L, Das R, Almahdi B, Curran HV, Morgan CJ (2011) Changes in cue reactivity and attentional bias following e x perimental cue e x posure and response prevention: a laboratory study of the effects of D-cycloserine in heavy drinkers. Psychopharmacology (Berl) 217:25–37.

Kennedy AP, Gross RE, Whitfield N, Drexler KP, Kilts CD (2012) A controlled trial of the adjunct use of D-cycloserine to facilitate cognitive behavioral therapy outcomes in a cocaine-dependent population. Addict Behav 37:900–907.

Klumpers F, Denys D, Kenemans JL, Grillon C, van der Aart J, Baas JM (2012) Testing the effects of Delta9-THC and D-cycloserine on e x tinction of conditioned fear in humans. J Psychopharmacol 26:471–478.

Krystal JH, Petrakis IL, Limoncelli D, Nappi SK, Trevisan L, Pittman B, D'Souza DC, Suckow RF (2011) Characterization of the interactive effects of glycine and D-cycloserine in men: further evidence for enhanced NMDA receptor function associated with human alcohol dependence. Neuropsychopharmacology 36:701–710.

Kuo MF, Unger M, Liebetanz D, Lang N, Tergau F, Paulus W, Nitsche MA (2008) Limited impact of homeostatic plasticity on motor learning in humans. Neuropsychologia 46:2122–2128.

Kuriyama K, Honma M, Koyama S, Kim Y (2011a) D-cycloserine facilitates procedural learning but not declarative learning in healthy humans: a randomized controlled trial of the effect of D-cycloserine and valproic acid on overnight properties in the performance of non-emotional memory tasks. Neurobiol Learn Mem 95:505–509.

Kuriyama K, Honma M, Yoshiike T, Kim Y (2013) Valproic acid but not D-cycloserine facilitates sleep-dependent offline learning of e x tinction and habituation of conditioned fear in humans. Neuropharmacology 64:424–431.

Kuriyama K, Honma M, Soshi T, Fujii T, Kim Y (2011b) Effect of D-cycloserine and valproic acid on the extinction of reinstated fear-conditioned responses and habituation of fear conditioning in healthy humans: a randomized controlled trial. Psychopharmacology (Berl) 218:589–597.

Kuriyama K, Honma M, Shimazaki M, Horie M, Yoshiike T, Koyama S, Kim Y (2011c) An N-methyl-D-aspartate receptor agonist facilitates sleep-independent synaptic plasticity associated with working memory capacity enhancement. Sci Rep 1:127.

Kushner MG, Kim SW, Donahue C, Thuras P, Adson D, Kotlyar M, McCabe J, Peterson J, Foa EB (2007) D-cycloserine augmented e x posure therapy for obsessive-compulsive disorder. Biol Psychiatry 62:835–838.

Litz BT, Salters-Pedneault K, Steenkamp MM, Hermos JA, Bryant RA, Otto MW, Hofmann SG (2012) A randomized placebo-controlled trial of D-cycloserine and e x posure therapy for posttraumatic stress disorder. J Psychiatr Res 46:1184–1190.

Matai x -Cols D, Turner C, Monzani B, Isomura K, Murphy C, Krebs G, Heyman I (2014) Cognitive-behavioural therapy with post-session D-cycloserine augmentation for paediatric obsessive-compulsive disorder: pilot randomised controlled trial. Br J Psychiatry 204:77–78.

Nadeau SE, Davis SE, Wu SS, Dai Y, Richards LG (2014) A pilot randomized controlled trial of d-cycloserine and distributed practice as adjuvants to constraint-induced movement therapy after stroke. Neurorehabil Neural Repair 28:885–895.

Nave AM, Tolin DF, Stevens MC (2012) Exposure therapy, D-cycloserine, and functional magnetic resonance imaging in patients with snake phobia: a randomized pilot study. J Clin Psychiatry 73:1179–1186.

Nesic J, Duka T, Rusted JM, Jackson A (2011) A role for glutamate in subjective response to smoking and its action on inhibitory control. Psychopharmacology (Berl) 216:29–42.

Nitsche MA, Jaussi W, Liebetanz D, Lang N, Tergau F, Paulus W (2004) Consolidation of human motor cortical neuroplasticity by D-cycloserine. Neuropsychopharmacology 29:1573–1578.

Ogawa M, Shigeto H, Yamamoto T, Oya Y, Wada K, Nishikawa T, Kawai M (2003) D-cycloserine for the treatment of ata x ia in spinocerebellar degeneration. J Neurol Sci 210:53–56.

Oliveto A, Benios T, Gonsai K, Feingold A, Poling J, Kosten TR (2003) D-cycloserine-Nalo x one interactions in opioid-dependent humans under a novel-response nalo x one discrimination procedure. E x p Clin Psychopharmacol 11:237–246.

Onur OA, Schlaepfer TE, Kukolja J, Bauer A, Jeung H, Patin A, Otte DM, Shah NJ, Maier W, Kendrick KM, Fink GR, Hurlemann R (2010) The N-methyl-D-aspartate receptor co-agonist D-cycloserine facilitates declarative learning and hippocampal activity in humans. Biol Psychiatry 67:1205–1211.

Otto MW, Basden SL, McHugh RK, Kantak KM, Deckersbach T, Cather C, Goff DC, Hofmann SG, Berry AC, Smits JA (2009) Effects of D-cycloserine administration on weekly nonemotional memory tasks in healthy participants. Psychother Psychosom 78:49–54.

Otto MW, Tolin DF, Simon NM, Pearlson GD, Basden S, Meunier SA, Hofmann SG, Eisenmenger K, Krystal JH, Pollack MH (2010) Efficacy of d-cycloserine for enhancing response to cognitive-behavior therapy for panic disorder. Biol Psychiatry 67:365–370.

Posey DJ, Kem DL, Swiezy NB, Sweeten TL, Wiegand RE, McDougle CJ (2004) A pilot study of D-cycloserine in subjects with autistic disorder. Am J Psychiatry 161:2115–2117.

Price KL, McRae-Clark AL, Saladin ME, Maria MM, DeSantis SM, Back SE, Brady KT (2009) D-cycloserine and cocaine cue reactivity: preliminary findings. Am J Drug Alcohol Abuse 35:434–438.

Price KL, Baker NL, McRae-Clark AL, Saladin ME, Desantis SM, Santa Ana EJ, Brady KT (2013) A randomized, placebo-controlled laboratory study of the effects of D-cycloserine on craving in cocaine-dependent individuals. Psychopharmacology (Berl) 226:739–746.

Prisciandaro JJ, Myrick H, Henderson S, McRae-Clark AL, Brady KT (2013a) Prospective associations between brain activation to cocaine and no-go cues and cocaine relapse. Drug Alcohol Depend 131:44-49.

Prisciandaro JJ, Myrick H, Henderson S, McRae-Clark AL, Santa Ana EJ, Saladin ME, Brady KT (2013b) Impact of DCS-facilitated cue e x posure therapy on brain activation to cocaine cues in cocaine dependence. Drug Alcohol Depend 132:195–201.

Randolph C, Roberts JW, Tierney MC, Bravi D, Mouradian MM, Chase TN (1994) D-cycloserine treatment of Alzheimer disease. Alzheimer Dis Assoc Disord 8:198–205.

Ressler KJ, Rothbaum BO, Tannenbaum L, Anderson P, Graap K, Zimand E, Hodges L, Davis M (2004) Cognitive enhancers as adjuncts to psychotherapy: use of D-cycloserine in phobic individuals to facilitate e x tinction of fear. Arch Gen Psychiatry 61:1136–1144.

Rodebaugh TL, Levinson CA, Lenze EJ (2013) A high-throughput clinical assay for testing drug facilitation of e x posure therapy. Depress An x iety 30:631–637.

Rosse RB, Fay-McCarthy M, Kendrick K, Davis RE, Deutsch SI (1996) D-cycloserine adjuvant therapy to molindone in the treatment of schizophrenia. Clin Neuropharmacol 19:444–450.

Rothbaum BO, Price M, Jovanovic T, Norrholm SD, Gerardi M, Dunlop B, Davis M, Bradley B, Duncan EJ, Rizzo A, Ressler KJ (2014) A randomized, double-blind evaluation of D-cycloserine or alprazolam combined with virtual reality e x posure therapy for posttraumatic stress disorder in Iraq and Afghanistan War veterans. Am J Psychiatry 171:640–648.

Santa Ana EJ, Rounsaville BJ, Frankforter TL, Nich C, Babuscio T, Poling J, Gonsai K, Hill KP, Carroll KM (2009) D-Cycloserine attenuates reactivity to smoking cues in nicotine dependent smokers: a pilot investigation. Drug Alcohol Depend 104:220–227.

Scheeringa MS, Weems CF (2014) Randomized placebo-controlled D-cycloserine with cognitive behavior therapy for pediatric posttraumatic stress. J Child Adolesc Psychopharmacol 24:69–77.

Scholl J, Gunthner J, Kolling N, Favaron E, Rushworth MF, Harmer CJ, Reinecke A (2014) A Role Beyond Learning for NMDA Receptors in Reward-Based Decision-Making-a Pharmacological Study Using d-Cycloserine. Neuropsychopharmacology 39:2900–2909.

Schwartz BL, Hashtroudi S, Herting RL, Schwartz P, Deutsch SI (1996) d-Cycloserine enhances implicit memory in Alzheimer patients. Neurology 46:420–424.

Siegmund A, Golfels F, Finck C, Halisch A, Rath D, Plag J, Strohle A (2011) D-cycloserine does not improve but might slightly speed up the outcome of in-vivo e x posure therapy in patients with severe agoraphobia and panic disorder in a randomized double blind clinical trial. J Psychiatr Res 45:1042–1047.

Steinglass J, Sysko R, Schebendach J, Broft A, Strober M, Walsh BT (2007) The application of e x posure therapy and D-cycloserine to the treatment of anore x ia nervosa: a preliminary trial. J Psychiatr Pract 13:238–245.

Storch EA, Murphy TK, Goodman WK, Geffken GR, Lewin AB, Henin A, Micco JA, Sprich S, Wilhelm S, Bengtson M, Geller DA (2010) A preliminary study of D-cycloserine augmentation of cognitive-behavioral therapy in pediatric obsessive-compulsive disorder. Biol Psychiatry 68:1073–1076.

Storch EA, Merlo LJ, Bengtson M, Murphy TK, Lewis MH, Yang MC, Jacob ML, Larson M, Hirsh A, Fernandez M, Geffken GR, Goodman WK (2007) D-cycloserine does not enhance e x posure-response prevention therapy in obsessive-compulsive disorder. Int Clin Psychopharmacol 22:230–237.

Tart CD, Handelsman PR, Deboer LB, Rosenfield D, Pollack MH, Hofmann SG, Powers MB, Otto MW, Smits JA (2013) Augmentation of e x posure therapy with post-session administration of D-cycloserine. J Psychiatr Res 47:168–174.

Teo JT, Swayne OB, Rothwell JC (2007) Further evidence for NMDA-dependence of the after-effects of human theta burst stimulation. Clin Neurophysiol 118:1649–1651.

Tsai GE, Falk WE, Gunther J (1998) A preliminary study of D-cycloserine treatment in Alzheimer's disease. J Neuropsychiatry Clin Neurosci 10:224–226.

Tsai GE, Falk WE, Gunther J, Coyle JT (1999) Improved cognition in Alzheimer's disease with short-term D-cycloserine treatment. Am J Psychiatry 156:467–469.

Urbano M, Okwara L, Manser P, Hartmann K, Herndon A, Deutsch SI (2014) A trial of D-cycloserine to treat stereotypies in older adolescents and young adults with autism spectrum disorder. Clin Neuropharmacol 37:69–72.

van Berckel BN, Hijman R, van der Linden JA, Westenberg HG, van Ree JM, Kahn RS (1996) Efficacy and tolerance of D-cycloserine in drug-free schizophrenic patients. Biol Psychiatry 40:1298–1300.

van Berckel BN, Lipsch C, Timp S, Gispen-de Wied C, Wynne H, van Ree JM, Kahn RS (1997) Behavioral and neuroendocrine effects of the partial NMDA agonist D-cycloserine in healthy subjects. Neuropsychopharmacology 16:317–324.

van Berckel BN, Lipsch C, Gispen-de Wied C, Wynne HJ, Blankenstein MA, van Ree JM, Kahn RS (1998) The partial NMDA agonist D-cycloserine stimulates LH secretion in healthy volunteers. Psychopharmacology (Berl) 138:190–197.

van Berckel BN, Evenblij CN, van Loon BJ, Maas MF, van der Geld MA, Wynne HJ, van Ree JM, Kahn RS (1999) D-cycloserine increases positive symptoms in chronic schizophrenic patients when administered in addition to antipsychotics: a double-blind, parallel, placebo-controlled study. Neuropsychopharmacology 21:203–210.

Watson BJ, Wilson S, Griffin L, Kalk NJ, Taylor LG, Munafo MR, Lingford-Hughes AR, Nutt DJ (2011) A pilot study of the effectiveness of D-cycloserine during cue-e x posure therapy in abstinent alcohol-dependent subjects. Psychopharmacology (Berl) 216:121–129.

Wilhelm S, Buhlmann U, Tolin DF, Meunier SA, Pearlson GD, Reese HE, Cannistraro P, Jenike MA, Rauch SL (2008) Augmentation of behavior therapy with D-cycloserine for obsessive-compulsive disorder. Am J Psychiatry 165:335–341; quiz 409.

Yoon JH, Newton TF, Haile CN, Bordnick PS, Fintzy RE, Culbertson C, Mahoney JJ III, Hawkins RY, Labounty KR, Ross EL, Aziziyeh AI, De La Garza R II (2013) Effects of D-cycloserine on cue-induced craving and cigarette smoking among concurrent cocaine- and nicotine-dependent volunteers. Addict Behav 38:1518–1526.

Yurgelun-Todd DA, Coyle JT, Gruber SA, Renshaw PF, Silveri MM, Amico E, Cohen B, Goff DC (2005) Functional magnetic resonance imaging studies of schizophrenic patients during word production: effects of D-cycloserine. Psychiatry Res 138:23–31.
